# Regulation of action potential delays via voltage-gated potassium Kv1.1 channels in dentate granule cells during hippocampal epilepsy

**DOI:** 10.3389/fncel.2013.00248

**Published:** 2013-12-05

**Authors:** Florian Kirchheim, Stefanie Tinnes, Carola A. Haas, Michael Stegen, Jakob Wolfart

**Affiliations:** ^1^Cellular Neurophysiology, Department of Neurosurgery, University Medical Center FreiburgFreiburg, Germany; ^2^Faculty of Biology, University of FreiburgFreiburg, Germany; ^3^Experimental Epilepsy Research, Department of Neurosurgery, University Medical Center FreiburgFreiburg, Germany; ^4^Department of Biomedicine, Institute of Physiology, University of BaselBasel, Switzerland; ^5^Oscar Langendorff Institute of Physiology, University of RostockRostock, Germany

**Keywords:** hippocampus, KD, Kcna1, *shaker*-related, homeostatic plasticity, homeostasis

## Abstract

Action potential (AP) responses of dentate gyrus granule (DG) cells have to be tightly regulated to maintain hippocampal function. However, which ion channels control the response delay of DG cells is not known. In some neuron types, spike latency is influenced by a dendrotoxin (DTX)-sensitive delay current (I_D_) mediated by unidentified combinations of voltage-gated K^+^ (Kv) channels of the Kv1 family Kv1.1–6. In DG cells, the I_D_ has not been characterized and its molecular basis is unknown. The response phenotype of mature DG cells is usually considered homogenous but intrinsic plasticity likely occurs in particular in conditions of hyperexcitability, for example during temporal lobe epilepsy (TLE). In this study, we examined response delays of DG cells and underlying ion channel molecules by employing a combination of gramicidin-perforated patch-clamp recordings in acute brain slices and single-cell reverse transcriptase quantitative polymerase chain reaction (SC RT-qPCR) experiments. An *in vivo* mouse model of TLE consisting of intrahippocampal kainate (KA) injection was used to examine epilepsy-related plasticity. Response delays of DG cells were DTX-sensitive and strongly increased in KA-injected hippocampi; Kv1.1 mRNA was elevated 10-fold, and the response delays correlated with Kv1.1 mRNA abundance on the single cell level. Other Kv1 subunits did not show overt changes in mRNA levels. Kv1.1 immunolabeling was enhanced in KA DG cells. The biophysical properties of I_D_ and a delay heterogeneity within the DG cell population was characterized. Using organotypic hippocampal slice cultures (OHCs), where KA incubation also induced I_D_ upregulation, the homeostatic reversibility and neuroprotective potential for DG cells were tested. In summary, the AP timing of DG cells is effectively controlled via scaling of Kv1.1 subunit transcription. With this antiepileptic mechanism, DG cells delay their responses during hyperexcitation.

## Introduction

The timing of action potential (AP) output is central to neuronal information processing in general and particularly for hippocampus-dependent memory formation (Buzsaki, 2002). The output of dentate gyrus granule (DG) cells is sparse but influential and has to be tightly regulated to maintain hippocampal function and protect downstream CA3 pyramidal cells (Treves and Rolls, 1992; Jung and McNaughton, 1993; Henze et al., 2002). Output regulation occurs at mossy fiber terminals of DG cells (Geiger and Jonas, 2000) and via synaptic feedback inhibition (Lawrence and McBain, 2003). The latter is thought to implement a temporal “winner-take-all” mechanism: activated DG cells compete in a race to AP threshold and fast responders silence slow neighbors (De Almeida et al., 2009). The race to threshold may be decided by subthreshold voltage-gated K^+^ (Kv) channels which can efficiently delay AP generation. However, DG cells express many types of Kv channels (Beck et al., 1992, 1996, 1997; Francis et al., 1997; Grosse et al., 2000; Riazanski et al., 2001; Rhodes et al., 2004; Ruschenschmidt et al., 2006) and it is not clear which channels govern the response delay of DG cells.

The α-dendrotoxin (DTX)-sensitive Kv channels of the Kv1 family (Kv1.1–6) can influence spike latency in various cell types, hence the name delay current (I_D_) (Storm, 1988; Golding et al., 1999; Bekkers and Delaney, 2001; Dodson et al., 2002; Glazebrook et al., 2002; Guan et al., 2006; Miller et al., 2008). Because cloned homomeric Kv1.1, Kv1.2, and Kv1.6 channels are DTX-sensitive (Stuhmer et al., 1989; Grupe et al., 1990; Grissmer et al., 1994), it is likely that these subunits mediate I_D_. However, other possibilities exist and precise identification of native delay channels is rare. For DG cells it is also unclear whether DTX-sensitive currents have an exclusive presynaptic function or whether they also influence somatodendritic signal integration (Sheng et al., 1994; Wang et al., 1994; Rhodes et al., 1997; Monaghan et al., 2001; Riazanski et al., 2001; Wenzel et al., 2007). Finally, ion channel expression of mature DG cells can change during temporal lobe epilepsy (TLE) (Ruschenschmidt et al., 2006; Stegen et al., 2009, 2012; Young et al., 2009). To better understand the function of DG cells and their role in TLE, it is necessary to precisely identify the involved ion channel molecules and their functional impact under physiological and pathophysiological conditions.

In the present study, we characterized the functional consequences of epilepsy-related plasticity of I_D_ in DG cells and identified the underlying ion channel subunits. We used the intrahippocampal kainate (KA) TLE mouse model which reproduces chronic focal seizures and hippocampal sclerosis (Suzuki et al., 1997; Riban et al., 2002; Häussler et al., 2012) as well as an *in vitro* KA model (Routbort et al., 1999; Bausch and McNamara, 2004; Tinnes et al., 2011; Chai et al., 2013). In affected hippocampi, DG cells possessed increased response delays, increased levels of Kv1.1 protein, and the Kv1.1 mRNA quantity correlated with response delays on the single-cell level. These results provide strong evidence that Kv1.1 subunits are regulated on the transcriptional level in response to hyperexcitation and that Kv1 channels constitute a “built-in” anticonvulsive and neuroprotective mechanism to scale DG output.

## Materials and methods

### Animal procedures

All procedures were conducted in accordance with the guidelines of the most recent European Community Council Directive (2010/663/EU) on the protection of animals for scientific purposes, and were approved by the regional council and local animal welfare officer according to the German animal protection act (Tierschutzgesetz). Mice were held in a 12 h light–dark cycle at 21.5—22.5°C with food and water *ad libitum*. For this study, 78 male C57Bl/6N mice were sacrificed. To simulate TLE *in vivo*, we employed the intrahippocampal kainic acid (KA) model which has been demonstrated to reliably produce chronic spontaneous hippocampal seizures and hippocampal sclerosis (Suzuki et al., 1997; Riban et al., 2002; Häussler et al., 2012). The manifestation of status epilepticus was verified in all cases via occurrence of forelimb clonus, head clonus/bobbing, rearing, and tonic/clonic seizures. We investigated DG cells from the KA-injected (ipsilateral) sclerotic foci of KA-injected hippocampi (“KA cells”) and DG cells of naïve, uninjected mice (“naïve cells”). To save animals, we did not reproduce previous sham-operations, in which we had observed no differences in properties of DG cells between naïve and saline-injected mice (Young et al., 2009). Stereotaxic KA injection was performed as previously described (Young et al., 2009). Briefly, adult mice (age at surgery: 5.9 ± 0.2 weeks old, *n* = 55) were anaesthetized by intraperitoneal injection of ketamine, xylazine, and atropine (0.1, 5, and 0.1 mg/kg body weight, respectively). After fixation in a stereotaxic frame (Kopf, Tujunga, CA, USA), 50 nl of 20 mM KA (in 0.9% NaCl) were injected via microsyringe (Hamilton, Bonaduz, Switzerland) in the right dorsal hippocampus (coordinates: dorsoventral −1.9 mm, anteriorposterior −1.9 mm, and mediolateral −1.5 mm, relative to Bregma). For brain slice preparation adult mice (age in weeks: KA, 9.7 ± 0.3, *n* = 55; naïve, 9.1 ± 0.2, *n* = 23) were anaesthetized with isoflurane and killed by decapitation. The brain was removed in ice-cold artificial cerebrospinal fluid (ACSF) containing (in mM): 87 NaCl, 25 NaHCO_3_, 2.5 KCl, 1.25 NaH_2_PO_4_, 0.5 CaCl_2_, 7 MgCl_2_, 75 sucrose and 10 glucose (equilibrated with 95% O_2_–5% CO_2_). Coronal slices (350 μm thick) containing dorsal hippocampi were collected with a vibratome (VT1200S, Leica, Bensheim, Germany), incubated for 30 min at 36°C and subsequently kept at room temperature (22–25°C) in sucrose ACSF until electrophysiological experiments.

### Electrophysiology

Patch-clamp recordings were performed at room temperature from DG cells of the suprapyramidal DG cell blade visualized by Dodt gradient contrast (Luigs and Neumann, Ratingen, Germany) video microscopy combining a 63 ×/1.0 or 40×/0.9 objective with a CCD camera (Axioskop2 FS, AxioCam ICm1, Zeiss, Oberkochen, Germany). In the recording chamber, slices were superfused with ACSF containing (in mM): 125 NaCl, 25 NaHCO_3_, 2.5 KCl, 1.25 NaH_2_PO_4_, 2 CaCl_2_, 1 MgCl_2_, and 25 glucose (equilibrated with 95% O_2_–5% CO_2_). Patch pipettes were pulled from borosilicate glass (Hilgenberg, Malsfeld, Germany, 2 mm outer and 1 mm inner diameter) using a DMZ-universal puller (Zeitz, Martinsried, Germany). Electrical signals were recorded using the amplifier SEC05LX (NPI, Tamm, Germany), an ITC18 D/A converter (Instrutech, Port Washington, NY, USA) and PatchMaster software (Heka, Lambrecht, Germany). Recordings were filtered at 20 kHz (voltage) and 2 kHz (current), and digitized at 20 kHz (voltage clamp) and 40–50 kHz (current clamp). Patch pipettes were filled with a solution containing (in mM): 135 potassium methylsulfonate (KMeSO_4_, “KMe”), 20 KCl, 10 HEPES, 0.1 EGTA, 2 MgCl_2_, 2 Na_2_ATP, and 0.2% biocytin (pH = 7.20). For recordings with subsequent molecular biology we used a KCl-based pipette solution containing (in mM): KCl 140, MgCl_2_ 3, HEPES 5, EGTA 5 (pH 7.35). For gramicidin-perforated patch recordings we used 40 mg gramicidin dissolved in 1 ml dimethylsulfoxide (DMSO) and kept at 7°C until dilution to final 40–80 μg/ml pipette solutions. Perforation by gramicidin was accepted when series resistance (*R*_ser_) was <40 MΩ, APs were overshooting, and resting membrane potential (*V*_rest_) below −73 mV. To ameliorate the stability of perforated recordings, we examined patch stability with different pipette solutions (see Supplementary Material and Figures S1A–C). As no differences were detected between KCl and KMe-based perforated delay recordings (naïve, KMe vs. KCl, *p* = 0.35; KA, KMe vs. KCl, *p* = 0.58), these data were pooled. If not noted otherwise KMe data was used. Seal resistances (*R*_seal_) of all recordings were >1GΩ (*R*_seal_: KMe, 4.4 ± 0.4 GΩ, *n* = 95; KCl, 5.5 ± 0.6 GΩ, *n* = 64). The *R*_ser_ was determined and compensated via bridge balance (*R*_ser_: KMe, 32.8 ± 0.6 MΩ, *n* = 91; KCl, 37.8 ± 0.6 MΩ, *n* = 61). In voltage-clamp, *R*_ser_ was either circumvented by using the switched mode of the SEC05LX amplifier when absolute current amplitudes were compared (switching frequencies 35–45 kHz), or it was compensated in the linear mode by 19–74% (*R*_ser_: naïve 39 ± 5%; KA, 41 ± 4%). The pipette capacitance was reduced electronically via the amplifier. A liquid junction potential of 10 mV was subtracted offline, except for KCl recordings. Only recordings were the input resistance (*R*_in_) reached a steady state and with *R*_in_/*R*_seal_ ratios of less than 0.3 were accepted. The passive properties of recorded mature DG cells of naïve mice and KA-injected mice were similar to those previously measured in whole-cell conditions (Young et al., 2009). The *V*_rest_ and AP amplitudes were not significantly different between naïve and KA cells (*V*_rest_: naïve, −82.4 ± 1.4 mV, *n* = 35; KA, −83.8 ± 0.9 mV, *n* = 60, *p* = 0.46; AP amplitudes: naïve, 63.7 ± 2.9 mV, *n* = 33; KA, 61.1 ± 2.3 mV, *n* = 53, *p* = 0.5). Absolute *R*_in_ values were elevated in perforated vs. whole-cell recordings, likely due to the higher *R*_ser_; however, the previously described relative *R*_in_ difference between naïve and KA cells recorded in whole-cell mode (Young et al., 2009) was conserved in perforated mode (*R*_in_: naïve, 602 ± 31 MΩ, *n* = 33; KA, 449 ± 27 MΩ, *n* = 56, *p* < 0.001). For synaptic bipolar voltage stimulation, theta glass pipettes (Hilgenberg) were pulled to tip sizes of ~10–50 μm and filled with ACSF solution. Lateral perforant path stimulation in the outer molecular layer (oML) was confirmed by paired-pulse-facilitation protocols in voltage-clamp (ratio last/first response, 1.4 ± 0.2, *n* = 9). In these stimulations (5 pulses at 100 Hz per trial), the first EPSP amplitude was used as input strength per trial and the AP number per trial was evaluated as output.

### Pharmacology

During recordings, the following inhibitors of AMPA/kainate-type and NMDA-type glutamate receptors, as well as GABA-A receptors were present in the ACSF: 50 μM D(-)-2-amino-5-phosphonopentanoic acid (D-AP5), 20 μM 1,2,3,4-tetrahydro-7-nitro-2,3-dioxoquinoxaline-6-carbonitrile disodium (CNQX), and 100 μM picrotoxin (PTX). During voltage-clamp experiments, we additionally added inhibitors of voltage-gated Na^+^ channels (tetrodotoxin, TTX, 0.5 μM) and K^+^ currents not studied here (forskolin, 50 μM; XE991, 10 μM). The Kv1 channel inhibitors α-dendrotoxin (DTX, 100 nM, see Introduction) and 4-aminopyridine, (4-AP, 40 μM) were applied when indicated. Drugs were kept in H_2_O stocks at −20°C (D-AP5, CNQX, TTX, forskolin) or in DMSO stocks at −20°C (PTX), diluted 1:1000 in ACSF and bath applied carbogen pressurized at 1.3–1.6 bar via an application system (AutoMate Scientific, Berkeley, CA, USA). We obtained D-AP5, CNQX, and TTX from Ascent Scientific (Weston-Super-Mare, U.K), DTX from Alomone (Jerusalem, Israel), and all other substances from Sigma-Aldrich (Munich, Germany).

### Immunocytochemistry

For Kv1.1 immunofluorescence, mice were transcardially perfused with paraformaldehyde [4% in 0.1 M phosphate buffer (PB), pH 7.4] 20 days after KA injection and brains were cryoprotected overnight in 20% saccharose in 0.1 M PB and frozen at −80°C. Mounted brains (TissueTech, Leica) were cut into 50 μm thin coronal slices with a cryotome (Leica) and washed in PB 4 times for 5 min at 4°C. A blocking solution [0.3% Triton X-100 plus 10% normal goat serum (NGS, Vector Laboratories, Burlingame, CA, USA)] was applied for 1 h at 4°C. Slices were incubated overnight in blocking solution containing the primary antibody (15 μg/ml anti-Kv1.1 mouse monoclonal antibody, clone K20/78, 75-007, UC Davis/NIH NeuroMab Facility, Davis, CA, USA). After washing as above, the secondary antibody was applied for 1 h at 4°C in PB (goat-anti-mouse-Cy3 1:200, Dianova, Hamburg, Germany). Thereafter sections were light protected and washed 3 times for 5 min at 4°C, in blocking solution, 0.1 M, and 0.005 M PB, respectively. Slices were mounted on gelatine-coated glass slides (Langenbrink, Emmendingen, Germany), air dried and coverslipped with ProLong gold (Invitrogen, Darmstadt, Germany).

### Reverse transcriptase quantitative polymerase chain reaction (RT-qPCR)

We performed RT qPCR at three levels of specificity: (i) microdissected DG cell layer, (ii) a pseudo single cell (SC) “pearl” RT-qPCR method, and (iii) a “true” SC RT-qPCR technique. The term pearl was used, because a string of ~10 DG cell bodies (= one sample) was harvested in a pearl-like fashion under visual control into a pipette with tip diameter slightly larger than the somata (Durand et al., 2006). For the true SC RT-qPCR technique, the cytosol of only a single neuron was harvested into the recording patch pipette and cells were subjected separately to qPCR (Stahlberg and Bengtsson, 2010). The SC RT-qPCR procedures were carried out under RNase-free conditions, i.e., using baked glassware (220°C, at least 4 h) and autoclaved plastic labware previously treated with 0.1% v/v diethyl dicarbonate (DEPC) water. For sample collection, patch pipettes were tip-filled with KCl-based intracellular solution using RNase-free water and chemicals containing gramicidin (see above) and backfilled with 6 μl KCl-intracellular solution without gramicidin. Because the KCl-based solution does not allow recording of the I_D_ under physiological conditions, we established a combination of perforated and SC RT-qPCR techniques: following electrophysiological recordings, the perforated mode was transformed into whole-cell mode and the cytosol was aspirated into the patch pipette while monitoring cell morphology and *R*_seal_. After 2–4 min of harvesting the cell was left in outside-out mode to avoid contamination with extracellular fluid. The pipette was washed twice in bath solution and its content was expelled into a 0.5 ml reaction vessel (Eppendorf) containing 14 μl of adjusted RT-buffer resulting in final concentrations of (in mM): KCl 75, MgCl2 3, Tris-HCl 50, DTT 5 and stored at −80°C. For cell lysis, this mix was briefly bath sonicated and the following components were added for a final volume of 20 μl (in mM): dNTP-mix 5, oligo(dT)-primer 0.005, random hexamer primer 0.005; supplemented with RNaseOut 40 U, SuperScriptIII RT 100 U. This RT mixture was incubated at 25°C for 5 min and at 37°C for 1 h and the enzyme was inactivated at 70°C for 15 min. Isolation of RNA from whole dissected dentate gyrii was performed with the RNeasy Mini Kit (Qiagen) according to the manufactures' instruction. As negative controls, identical procedures were performed (i) with water instead of RT enzyme, (ii) without cell sample, and (iii) with aspirated ACSF instead of harvested cytosol. Quantitative PCR was conducted using an iCycler real-time PCR machine (BioRad, München, Germany). The reaction was conducted in a 20 μ l volume containing 10 μ l of 2× SYBR Green master mix (Applied Biosystems, Warringten, U.K.), 2.5 μ l of sample cDNA (6 μl for organotypic hippocampal slice cultures, OHCs), 1 μ l of diluted gene specific primer set and nuclease-free water. Cycle threshold (CT) values of product detection were determined using the IQ5 software (BioRad). The enzyme glyceraldehyde 3-phosphate dehydrogenase (GAPDH) was used as positive RT control and cells not expressing GAPDH were not further analyzed (GAPDH CTs, SC RT-qPCR: naïve, 34.8 ± 0.9, *n* = 24; KA, 34.9 ± 0.6, *n* = 36; *p* = 0.95). For employed primer sequences see Supplementary Material. The thermal profile included 15 min at 95°C, 50 cycles at 95°C for 15 s, 60°C for 15 s, and 72°C for 30 s. Melting curve analysis was performed on each sample to test product size and primer dimer formation. Only samples with a single product-specific peak and no primer dimer were further analyzed. Each assay was also validated on agarose gels stained with GelRed 1:10000 (Biotium, Hayward, CA, USA). Primer efficiencies (*E*, between 0.9 and 1.05) were obtained by preparing at least five 10-fold dilutions of whole brain cDNA as a template for qPCR, fitting the slope of the linear regression of CT values vs. log^10^ of template concentrations, and calculating *E* = 10^∧^(−1/slope).

### Organotypic hippocampal slice cultures (OHCs)

In order to test the reversibility of DG cell changes we used an established *in vitro* seizure model which leads to increased DG cell activation and other cellular symptoms of TLE (Routbort et al., 1999; Bausch and McNamara, 2004; Tinnes et al., 2011; Chai et al., 2013). The OHCs were prepared from C57Bl6/N mice (P2-P4) as described for rats (Tinnes et al., 2011). Briefly, hippocampi were aseptically microdissected and cut into 400 μm thick transverse sections using a McIlwain tissue chopper (Mickle, Goose Green, UK). Slices were cultivated for 7 days *in vitro* (DIV) on Millicell membranes (pore 0.4 μm, diameter 30 mm, Millipore, Tullagreen, Ireland) in 5% CO2 at 37°C in nutrition medium (46% minimal essential medium, 25% basal medium Eagle, 25% heat-inactivated horse serum with 0.65% glucose and 2 mM glutamine, pH 7.2). The medium was changed every 48 h. On the 8th DIV, OHCs were treated for 24 h with 10 μM KA and thereafter kept in nutrition medium. In a subset of wells, KA was applied with DTX (100 nM) which thereafter was added freshly with every medium change. Controls were incubated with nutrition medium only. For electrophysiological experiments, Millicell membranes with OHCs were cut out and transferred to the recording chamber. To monitor dying cells (see also data analysis), OHC slices were incubated with propidium iodide (PI, 5 μg/ml) for 20 min and fluorescence was imaged with an inverse microscope (CKX41, Olympus) equipped with CACHN 10×/0.25 PHP objective, a fluorescence lamp (U-RFL-T), and filters (U-MWG2; excitation, 530–550 nm; dichromatic mirror, 570 nm; emission, 590 nm). Images were acquired with a CCD camera (E-450 SLR, Olympus) and manually assembled in Illustrator (Adobe, München, Germany).

### Data analysis

Electrophysiological records were analyzed using the software FitMaster (Heka) and IgorPro (WaveMetrics, Portland, OR, USA). At the beginning of each recording the following cellular parameters were collected in control ACSF (CTRL): *V*_rest_ (averaged from a ~25 s trace), *R*_in_ (as slope of the steady state current (I)-voltage (U) relation within ±10 mV from *V*_rest_), membrane time constant (τ*_m_*, fitting a double exponential function to the average of ~25 voltage responses of <5 mV from *V*_rest_, using only the slow component), cell capacitance (as τ*_m_*/*R*_0_ with *R*_0_ = U*_m_*/*I_m_*), current inactivation time constant (τ_inact_, fitting a mono exponential function to DTX-sensitive currents evoked with a 2.5 s pulse to 0 mV), rheobase (as minimum current needed to obtain at least one AP within 1 s), AP threshold (via voltage slope change >20 mV/s at rheobase), AP response delay (time from start of current injection to AP peak within 2 s trace), and AP width (measured at −5 mV). Voltage dependence of activation and inactivation was fitted by a Boltzmann function *G*/*G*_max_ (or *I*/*I*_max_) = 1/(1+exp((*V*_50_ − U*_m_*/*k*))) were *V*_50_ is the voltage of half-maximal activation (or inactivation) and *k* the slope factor. The reversal potential (*V*_rev_) was determined by a linear fit to DTX-sensitive peak currents evoked by 20 mV steps from −110 to +30 mV and calculation of the fits crossing point with the abscissa. For the analysis of relative mRNA expression levels we used the ΔΔCT-Method with GAPDH as reference (in dissected tissue) and absolute log^2^-transformed CT values (for SC). Correlation of subunit expression co-variances were analyzed via the z-score, i.e., by subtracting log^2^-transformed CT values from mean values of respective subunits and dividing by the SD. For offline image analysis in OHCs, a ROI was defined in each OHC which contained a clearly identifiable DG cell layer (Tinnes et al., 2011; Chai et al., 2013) and the mean RGB red channel pixel intensities were read out in Photoshop (Adobe). Signal densities were calculated by normalizing pixel intensities to ROI area. Sizes of ROIs were not different among compared groups (*p* = 0.37). For offline image analysis of Kv1.1 stainings, columnar ROIs from hilus to fissure were selected and the red signal was normalized to the red intensity of contralateral DG-fissure using MultiGauge v3.0 (Fujifilm, Düsseldorf, Germany). Statistical significance of group differences was assessed with the software Prism (GraphPad, San Diego, CA, USA) applying the following tests: Shapiro-Wilk normality test (to verify normal distribution), Mann–Whitney's test (for 2 groups not normally distributed), Students *t*-tests (for 2 groups normally distributed), and the *F*-test (for regressions and curve comparisons). Significance of correlation was determined according to a table of Pearson's *r*-values. Levels of significance are indicated in figures as *(<0.05), **(<0.01), and ***(<0.001). Arithmetic mean values are ± s.e.m. and numbers represent cells if not mentioned otherwise. Figures were produced using IgorPro, Prism, and Illustrator.

## Results

### The functional impact of DTX-sensitive action potential response delays in DG cells of naïve mice and during hippocampal epilepsy

**Figure 1:**
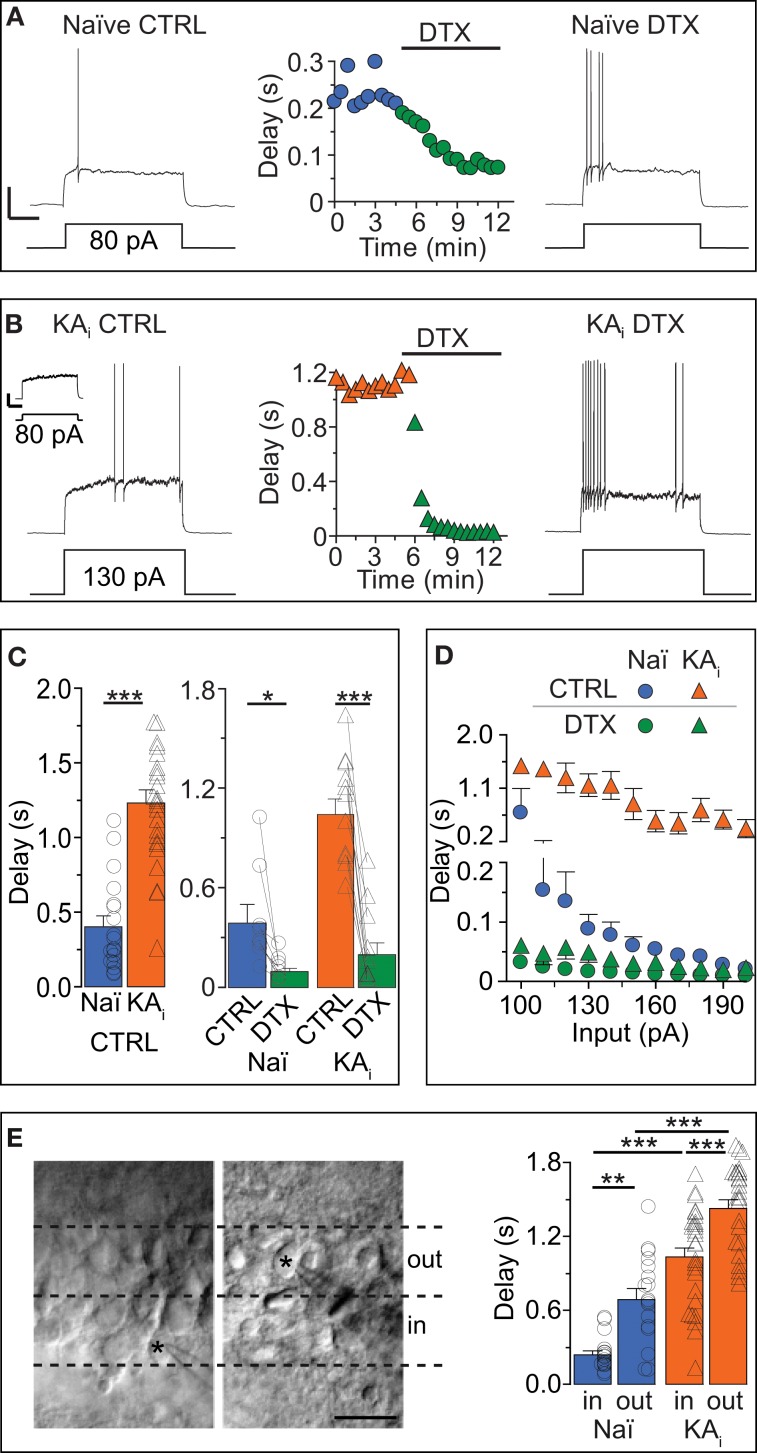
**Dendrotoxin (DTX)-sensitive action potential response delays in DG cells of naïve mice and during hippocampal epilepsy. (A–C)** Current-clamp recordings in DG cells of naïve [*Naï*, **(A)**, *circles*] and KA-injected [*KA*_i_, **(B)**, *triangles*] mice showing the transformation of a delayed action potential (AP) response under control condition (*CTRL*, *left traces*) into an almost immediate AP response by Kv1 channel blocker *DTX (right traces)*. Because KA DG cells have a higher rheobase than Naï DG cells (Young et al., 2009), stronger current steps were injected into KA cells for pharmacological delay characterization [130 pA in **(B)**]. For direct comparison see *inset* in **(B)** [response of KA cell to current pulse as in **(A)**, i.e., 80 pA]; *Scale bars* in **(A,B)** *inset*, 25 mV, 0.5 s. Compared to Naï DG cells, AP delays of KA DG cells were ~2.5 times prolonged **(C)**. This was true with rheobase currents **(C**, *right panel***)** and with current injections that were not statistically different **(C**, *left panel***)**. **(D)** Over a larger range of current injections, the response delays of KA DG cells were elevated (*orange triangles*) vs. Naï DG cells (*blue circles*) which displayed stronger response acceleration with increasing current injections. This difference was abolished with DTX (*green triangles*, KA; *green circles,* Naï, respectively). **(E)** Recorded DG cells localized via live fotos of patch pipettes (asterisks) were devided into inner DG cells (“*in*,” *left panel*, i.e., cells closer to the hilus outer) and outer DG cels (“*out*,” i.e., cells closer to the molecular layer). The grouping of recorded AP delays according to these areas (*right panel*) revealed on average longer delays in the outer DG cells from both naïve and KA-injected mice.

Our first question was: which current controls the AP response delay of DG cells? We depolarized DG cells with a step current above rheobase and measured the AP delay. To avoid current rundown, all recordings were performed in the gramicidin-perforated patch configuration for which we established an improved stability method (see Methods and Figures S1A–C in Supplementary Material). The passive and morphological properties of recorded mature DG cells of naïve mice (“naïve cells”) and of epileptic, KA-injected mice (“KA cells”) were similar to those previously recorded under whole-cell conditions (Young et al., 2009) (Figures 1A,B; also see Methods). The AP delays of naïve DG cells were sensitive to 40 μM 4-AP (data not shown) and 100 nM DTX, an inhibitor of Kv channels of subtypes Kv1.1, Kv1.2, and Kv1.6 (see Introduction; Figures 1A,C) (paired test, delay naïve: CTRL, 390 ± 106 ms; DTX, 98 ± 24 ms, *n* = 8, *p* < 0.05). Thus, the answer to the first question is: the response delay of DG cells is controlled via a DTX-sensitive current, reminiscent of the delay current (I_D_) of other cell types (see Introduction).

The next question we addressed was: does the response delay of DG cells change in conditions of hippocampal epilepsy and how does it affect the excitability of DG cells? We recorded DG cells of mice with intrahippocampal KA injection (see Methods). This TLE model has been previously proven to reliably produce chronic hippocampal seizures and many other symptoms associated with TLE including hippocampal sclerosis (Suzuki et al., 1997; Riban et al., 2002; Young et al., 2009; Häussler et al., 2012). The KA cells of sclerotic hippocampi possessed ~3-times longer AP delays compared to naïve cells (delays: naïve, 402 ± 73 ms, *n* = 18; KA, 1237 ± 89 ms, *n* = 25, *p* < 0.001; Figures 1A–C). Note that for the pharmacological characterization of delays, we used different direct current (DC) pulses in naive and KA cells (Figures 1A,B) but for the statistical delay comparison we used only recordings with similar DC pulses (DC: naïve, 81 ± 6 pA, *n* = 18; KA, 90 ± 8 pA, *n* = 25, *p* = 0.34). The DTX sensitivity of response delays of KA cells was very prominent (paired test, delay KA: CTRL, 1046 ± 96 ms; DTX, 201 ± 71 ms, *n* = 11, *p* < 0.01; Figures 1B,C) and delay differences between naïve and KA cells were not apparent in DTX (evaluated with similar DC pulses; delay in DTX: naïve, 114 ± 41 ms, *n* = 6; KA, 126 ± 78 ms, *n* = 6, *p* = 0.81). Application of DTX also had a minor effect on the *R*_in_ of KA DG cells indicating that I_D_ slightly contributes to the reduced *R*_in_ of KA cells previously discovered (Young et al., 2009) (paired tests, *R*_in_ naïve: CTRL, 541.1 ± 31.7 MΩ; DTX, 579.8 ± 22.6 MΩ, *n* = 9, *p* = 0.08; *R*_in_ KA: CTRL, 448.4 ± 22.6 MΩ; DTX, 499.9 ± 25.8 MΩ, *n* = 11, *p* < 0.05). However, inwardly rectifying K^+^ (Kir) channels were found mainly responsible for the *R*_in_ difference between naïve and KA cells (Young et al., 2009). Accordingly, the *R*_in_ difference remained in DTX (*R*_in_ DTX: naïve vs. KA, *p* < 0.05) and response delays of KA cells were not much affected by 0.2 mM barium, which blocks Kir channels (*n* = 12; data not shown). These results suggest a clear functional separation of Kv1 and Kir channels.

Since the data described above only show single input pulses, we additionally analyzed the delay over a broader range of current injections (Figure 1D). In naïve cells, current injections with increasing strength effectively shortened the response delays (Figure 1D, blue circles) which leveled off around ~10 ms with stronger injections. In contrast, delays of KA cells, although reduced with increasing current injections, saturated around a delay of ~200 ms and were not further reduced even with stronger injections (Figure 1D, orange triangles). Thus, the answer to the second question is: in a mouse model of TLE, the response delay of DG cells is markedly increased and consequently the excitability of DG cells is decreased. This decrease adds to the described *R*_in_ reduction which already diminishes the excitability via the rheobase (Young et al., 2009).

### Heterogeneous response delays: slowly responding DG cells are close to molecular layer

Little information exists about the heterogeneity of response phenotypes within the population of mature DG cells. We grouped our physiological data from naïve and KA mice into “inner” and “outer” cells, i.e., closer to the hilus and closer to the ML, respectively (Figure 1E). Consistent with previous reports (Liu et al., 2000), in naïve animals, *R*_in_ values showed a tendency toward lower values in outer DG cells compared to inner DG cells (*R*_in_ naïve: inner, 430.7 ± 50.1 MΩ, *n* = 16; outer, 380.8 ± 45.5 MΩ, *n* = 17, *p* = 0.46), a difference which became significant in KA cells (*R*_in_ KA: inner, 330.4 ± 28.9 MΩ, *n* = 27; outer, 258.6 ± 17.5 MΩ, *n* = 26, *p* < 0.05). It could be suspected that immature DG cells located in the subgranular zone were part of the inner population but none of the DG cells presented in the present study displayed any of the clearly identifiable properties of adult-born DG cells (Schmidt-Hieber et al., 2004; Häussler et al., 2012). We found that on average, DG cells located in the outer cell layer had longer response delays than DG cells lying in the inner layer (Figure 1E). This effect was true for naïve cells (delay naïve: inner, 251 ± 35 ms, *n* = 16; outer, 707 ± 88 ms, *n* = 17; *p* < 0.01), as well as for KA cells (delay KA: inner, 1048 ± 77 ms, *n* = 27; outer, 1453 ± 67 ms, *n* = 26; *p* < 0.001; with similar DC injections: naïve inner vs. outer, *p* = 0.48; KA inner vs. outer, *p* = 0.90). These results suggest that the population of mature DG cells is heterogeneous with respect to response speed.

### The input/output transfer function of DG cells is controlled by I_D_

**Figure 2:**
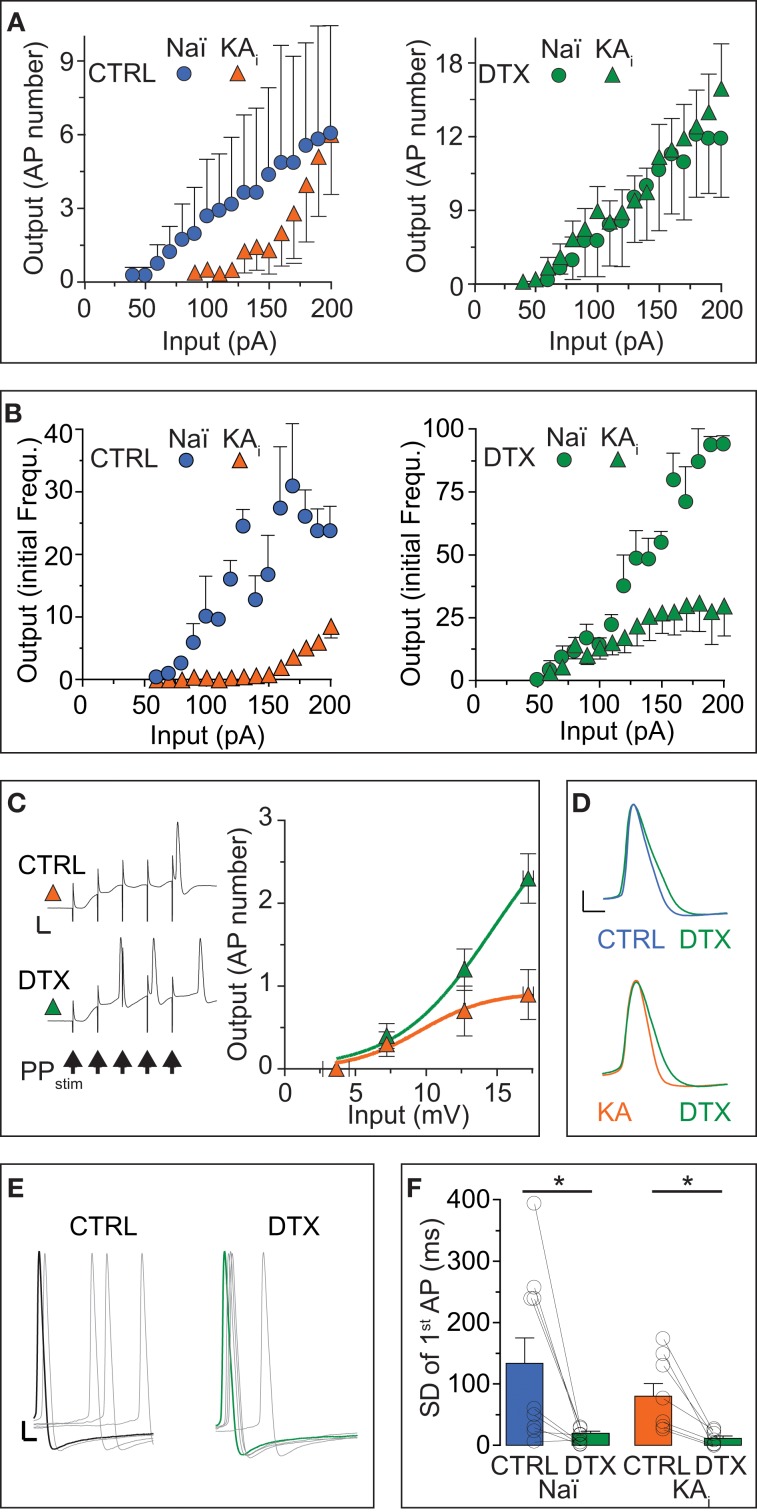
**The input/output transfer function of DG cells is controlled by I_D_. (A,B)** Action potential (AP) output evaluated by number **(A)** and initial frequency **(B)** was evoked by somatic DC input in DG cells from naïve mice (*Naï, circles*) and KA-injected mice (*KA, triangles*) under control (CTRL) conditions and after DTX application (*green symbols*). The input/output (I/O) curve was shifted to higher input values in KA vs. Naï cells [**(A)**, *left panel*, compare *orange triangles* with *blue circles*, respectively]. Application of DTX reduced this difference [**(A,B)**, *right panels*, compare *green triangles* with *green circles,* respectively], although with higher input, the AP frequency did not reach naïve levels. **(C)** To test the effect of increased DTX-sensitive conductance on dendritic signal integration, the above changes in I/O curves were additionally verified with extracellular perforant path stimulation (5 pulses at 100 Hz, *arrows* in *left panel*) evoking excitatory postsynaptic synaptic potentials (EPSPs) in KA cells of which the first was measured as *input* (no such additional verification was performed in naïve cells). Synaptic input triggered no more than one AP at the end of five summating EPSPs in CTRL conditions (upper trace and orange triangles, respectively; scale bars, 20 mV, 50 ms). However, application of DTX shifted the I/O curve to lower input values and allows multiple APs to occur (*lower trace, green triangles*). **(D–F)** Application of DTX increased the AP width **(D)** and 1st AP precision **(E,F)** of the AP response to DC steps in Naï and KA DG cells. The AP jitter evaluated as SD of first spike (see Results for CV). *Scale bars* in **(D)**, 10 mV, 1 ms; **(E)** 10 mV, 2 ms.

As another important measure of neuronal excitability, in addition to rheobase and response speed, the AP number and frequency within a response characterize the input/output (I/O) transformation of neurons. Therefore, we assessed the effect of DTX on the I/O curves of naïve and KA cells with respect to AP numbers. Naïve cells displayed relatively linear I/O curve in the tested range of somatic DC injections (Figure 2A, blue circles). In KA cells, the I/O function was shifted to higher input values and only large DCs evoked multiple APs (*F*-test, naïve vs. KA, *p* < 0.0001, *n* = 6 and 14, respectively; Figure 2A, orange triangles). The application of DTX not only increased the output of naïve and KA cells (naïve, *p* < 0.0001; KA, *p* < 0.0001) but strongly reduced the differences between naïve and KA I/O transfer functions (*p* = 0.38; Figure 2A, green circles and triangles). Because as another important function, the I_D_ can also influence the AP frequency (Miller et al., 2008), we constructed the I/O function of initial frequencies for DG cells (Figure 2B). This I/O curve again demonstrates the low excitability of KA vs. naïve cells (*F*-test, naïve (blue circles) vs. KA (orange triangles), *p* < 0.0001, *n* = 5 and 10, respectively). For lower input values, the difference in initial frequency was reduced with DTX (*F*-test, naïve, *p* < 0.0001; KA, *p* < 0.0001; Figure 2B, green circles and triangles). However, at higher input currents, a frequency reduction remained in KA cells even during DTX (Figure 2B, green circles and triangles). This effect is likely due an enlarged fast afterhyperpolarization in KA cells limiting the minimal interspike interval (Kirchheim, unpublished data). In summary, the I/O curves corroborate the above described decrease of excitability in KA vs. naïve cells due to the I_D_ upregulation and reveal the enormous functional impact of I_D_ on the I/O transformation in DG cells.

Although the above results already answered the question of how the I/O of DG cells is affect by I_D_ regulation, it is possible that the increased I_D_ does not affect the somatodendritic integration of synaptic inputs arriving at distal synapses. We tested this hypothesis in KA cells by focused stimulation of the lateral perforant path and evaluating the EPSP of a cumulative train of 5 EPSPs as input strength (Figure 2C). Under control conditions, it was difficult to obtain more than 1 AP from KA cells, even with maximal and prolonged synaptic stimulation (not shown): the I/O curve saturated already at low input values (Figure 2C, orange triangles). However, in DTX, bursts of 2–3 APs were frequently triggered by the synaptic input (Figure 2C, green triangles). These data demonstrate the strong influence of I_D_ upregulation on dendritic EPSP integration and on the sparseness of DG cell activation.

Many studies have shown that Kv1 channels influence the shape and precision of APs in the submillisecond range (Geiger and Jonas, 2000; Kole et al., 2007; Cudmore et al., 2010; Foust et al., 2011; Higgs and Spain, 2011). Although this is not the focus of the present study, we determined the effect of DTX on AP shape and precision in naïve and KA cells. Application of DTX slightly shifted the AP threshold to more hyperpolarized potentials (paired tests, AP threshold naïve: CTRL, −44.7 ± 3.2 mV; DTX, −47.9 ± 1.8 mV, *n* = 9, *p* = 0.13; AP threshold naïve KA: CTRL, −37.6 ± 1.6 mV; DTX, −42.0 ± 2.5 mV, *n* = 11, *p* = 0.11), such that respective differences between naïve and KA were reduced (AP threshold CTRL: naïve vs. KA, *p* < 0.05; DTX: naïve vs. KA, *p* = 0.08.). Furthermore, DTX prolonged the AP duration and this effect was more obvious in KA vs. naïve cells (paired test, AP width naïve: CTRL, 1.13 ± 0.05 ms; DTX, 1.25 ± 0.04 ms, *n* = 9, *p* < 0.05; AP width KA: CTRL, 1.09 ± 0.05 ms; DTX, 1.27 ± 0.05, *n* = 11, *p* < 0.05; Figure 2D). In addition, DTX decreased the absolute jitter (SD) of the 1st AP timing (paired tests, SD naïve: CTRL, 134 ± 41 ms; DTX, 20 ± 4 ms, *n* = 9 cells; *p* < 0.05; SD KA: CTRL, 80 ± 21 ms; DTX, 12 ± 4 ms, *n* = 7; *p* < 0.05; Figures 2E,F). However, when evaluating AP jitter relative to first spike delay (which increased during DTX as shown), i.e., as coefficient of variation (CV), DTX had no detectable effect (paired tests, CV naïve: CTRL, 0.65 ± 0.11; DTX, 0.74 ± 0.08, *n* = 9; *p* = 0.55; CV KA: CTRL, 0.74 ± 0.06; DTX, 0.86 ± 0.12, *n* = 7; *p* = 0.41). Thus, in addition to response delay, I_D_ also controls the AP shape and precision in DG cells. In summary, the results of this section show that the response phenotype of DG cells is profoundly influenced by DTX-sensitive I_D_, in particular in a TLE mouse model were DG cells show a strong increase of I_D_ and a decrease inexcitability.

### Voltage-clamp characterization of the I_D_ of DG cells.

**Figure 3:**
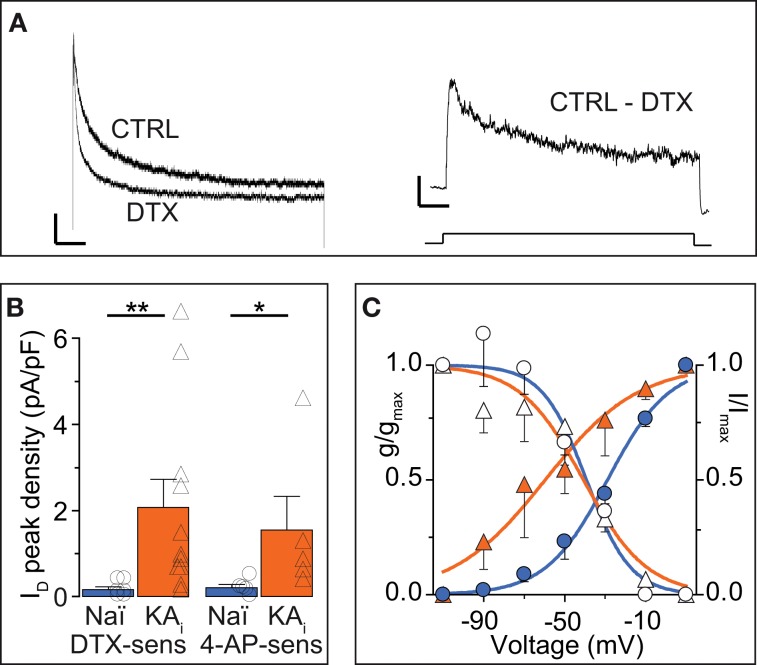
**Voltage-clamp characterization of the I_D_ of DG cells. (A)** Dendrotoxin (DTX, 100 nM)-sensitive currents (I_D_) were obtained in voltage-clamp by offline subtraction of currents after DTX application from those before (*CTRL*). *Scale bars*, 500 ms, *left panel*, 100 pA, *right panel*, 50 pA. *Lower panel*, 2.5 s-long voltage step from −100 to 0 mV). **(B)** Peak I_D_ current densities from experiments as in *A* using DTX and 40 μM 4-AP. The DG cells of KA-injected hippocampi had strongly increased DTX- and 40 μM 4-AP-sensitive I_D_ amplitudes. **(C)** To determine the voltage dependence of activation, the DTX-sensitive I_D_ was evoked by voltage steps from −110 to +10 mV (*filled symbols, left y-axis*), transformed into conductance (*g*) and a Boltzmann function was fitted [*V*_Act(0.5)_, naïve, ‒32.3 ± 1.9 mV, slope, 17.1, *n* = 7; KA, ‒46.0 ± 2.3 mV, slope, 17.0, *n* = 7, *p* < 0.001]. To determine voltage dependence of inactivation, prepulses from −110 to +10 mV (*empty symbols, right y-axis*) were applied prior to the test pulse (+10 mV) and normalized I_D_ amplitudes were fitted to a Boltzmann function [*V*_Inact(0.5)_: Naï, −41.7 mV, slope, 14.1; KA, −43.8 mV, slope, 13.7].

The I_D_ has been well-characterized in some cell types (Storm, 1988; Golding et al., 1999; Bekkers and Delaney, 2001; Dodson et al., 2002; Glazebrook et al., 2002; Guan et al., 2006; Miller et al., 2008) but not in DG cells. Therefore, and because hints for Kv channel subunit identity may be gained from these properties (see Discussion), we analyzed the biophysical characteristics of DTX-sensitive I_D_ obtained in voltage-clamp recordings (Figure 3A). Additional inhibitors were present during these recordings (see Methods). First it was verified that the I_D_ was indeed carried by K^+^ ions (*V*_rev_ of I_D_, −99.1 ± 15.1 mV, *n* = 10; K^+^ Nernst potential, −104.3 mV). The I_D_ of naïve cells recorded under these conditions was small, but clearly present and functionally relevant as demonstrated in the current-clamp measurements above (naïve: I_D_ at 0 mV, 14.9 ± 5.9 pA; I_D_ density, 0.17 ± 0.06 pA/pF, *n* = 7). In comparison, KA cells possessed a much larger I_D_ (KA: I_D_, 185.2 ± 65.2 pA; I_D_ density, 2.07 ± 0.66 pA/pF; *n* = 11; KA vs. naïve, *p* < 0.01; Figure 3B). Consistent with the 4-AP sensitivity of I_D_ (Storm, 1988), currents sensitive to 40 μM 4-AP were also enhanced in KA cells (Figure 3B). Interestingly, the voltage-dependence of activation (*V*_50act_) was shifted to more hyperpolarized values in KA cells (*V*_50act_: naïve, −32.3 ± 1.9 mV, *n* = 7; KA, −46.0 ± 2.3 mV, *n* = 7, *p* < 0.001; Figure 3C). The kinetics and voltage-dependence of inactivation (τ_inact_, *V*_50inact_, respectively) were not different in naïve and KA cells (τ_inact_: naïve, 250 ± 53 ms, *n* = 4; KA, 378 ± 103 ms, *n* = 5, *p* = 0.79; *V*_50inact_: naïve, −41.7 ± 1.5 mV, *n* = 7; KA, −43.8 ± 2.4 mV, *n* = 7, *p* = 0.59; Figure 3C). These voltage-clamp data demonstrate an increased I_D_ in KA vs. naïve cells and the shifted voltage-dependence points to a change in Kv subunit composition (Stuhmer et al., 1989; Grissmer et al., 1994).

### RT-qPCR reveals that Kv1.1 subunits mediate the I_D_ increase in DG cells during hippocampal epilepsy

**Figure 4:**
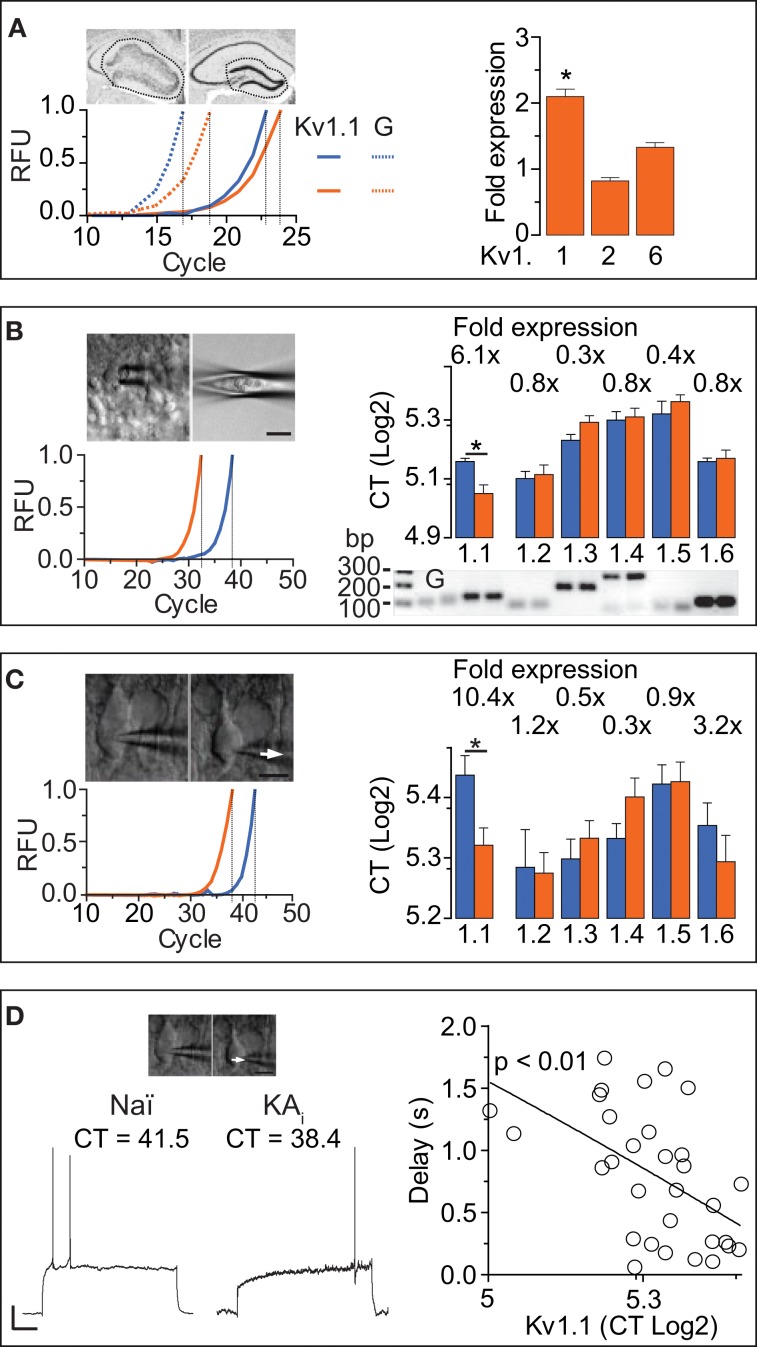
**RT-qPCR reveals that Kv1.1 channel subunits mediate the I_D_ increased in DG cells during hippocampal epilepsy. (A)** In a first RT-qPCR approach, whole DGs were dissected from acute brain slices and expression levels of Kv1.1, Kv1.2, and Kv1.6 were measured relative to GAPDH [*left upper panels:* scheme of dissection; *left lower panel, solid lines: red*, naïve; *blue*, KA; *dashed lines:* GAPDH; *RFU*, relative fluorescence units; traces normalized to cycle threshold (CT)]. In the *right panel*, the relative expression levels of Kv subunits were statistically compared between KA and control tissue (not shown). Kv1.1 was expressed ~2 fold in the epileptic DG, while Kv1.2, and Kv1.6 were not significantly different (in KA vs. control tissue). **(B)** DG cell somata were collected into the pipette in a “pearl” fashion (see Methods, left *upper panels*) and mRNA reverse transcribed to cDNA which was subjected to qPCR. Real time amplification of Kv1.1 (*left lower panel*; *blue*, naïve; *orange*, KA). The amplified PCR products of Kv1.1, Kv1.2, Kv1.3, Kv1.4, Kv1.5, and Kv1.6 subunits, and GAPDH *(G)* had the expected fragment sizes as revealed by quantitative gel electrophoresis (*right lower panel*; *bp*, base pairs). Relative to naïve DG cells, expression levels of Kv1.1 subunits were increased in KA samples, while Kv1.2, Kv1.3, Kv1.4, Kv1.5, and Kv1.6 abundance was not significantly changed (*right upper panel*). **(C,D)** In a second approach, true single-cell (SC) RT-qPCR experiments were performed from individual cell cytosol harvested (arrows) in patch-clamp recordings **(C**, *left upper panel***)**. Amplification and evaluation of Kv1.1–6 subunits in SC RT-qPCR **(C**, *left lower panel***)** was performed as in **(B)**. To circumvent the effect of the non-physiological pipette solution, gramicidin-perforated recordings **(D**, *left panel***)** were performed prior to membrane rupture. In these experiments, KA cells expressed ~10-fold more Kv1.1 subunits compared to naïve cells, while levels of other Kv1 subunits were again not different **(C**, *right panel***)**. Importantly, the AP response delays correlated with the levels of Kv1.1 expression in the respective pooled naïve (Naï) and KA cells [**(D)**, *right panel*; *r* = 0.98, *n* = 32, *p* < 0.01].

The DTX-sensitivity of response delays indicated the involvement of Kv1 channels. However, as DTX-insensitive subunits can form heteromultimeric channels with DTX-sensitive subunits, the precise molecular identity of the delay channels is unknown (see Discussion). Therefore, we employed RT-qPCR techniques to determine the Kv1 subunit mRNA underlying the delayed AP responses of DG cells. We performed the qPCR analysis on three different levels: (i) microdissected dentate gyrii (Figure 4A), (ii) multiple DG cell bodies harvested in a “pearl”-like fashion into the pipette (Figure 4B), and (iii) the true SC RT-qPCR method where single cytosols of recorded cells are harvested one-by-one (Figure 4C) (see Methods and Supplementary Material). In the microdissected tissue, Kv1.1, Kv1.2, and Kv1.6 were tested and detection thresholds (CT values) were normalized to GAPDH-expression. While Kv1.2 and Kv1.6 expression levels were not significantly different between naïve and KA samples, Kv1.1 mRNA was enhanced ~2-fold in KA vs. naïve samples (fold expression: Kv1.1, 2.1 ± 0.41, *p* = < 0.05; Kv1.2, 0.82 ± 0.88, *p* = 0.41; Kv1.6, 1.33 ± 0.26, *p* = 0.85; *n* = 14, respectively; Figure 4A).

In the next step, we applied the pearl method to all known Kv1 subunits (Figure 4B). In naïve samples, Kv1.1–6 subunits were present in the following relative CT order (lower CT values = higher abundance): Kv1.2 (34.4 ± 0.7, *n* = 10) > Kv1.6 (34.9 ± 0.3, *n* = 6) > Kv1.1 (35.9 ± 0.4, *n* = 8) > Kv1.3 (37.8 ± 0.5, *n* = 7) > Kv1.4 (39.6 ± 0.8, *n* = 8) > Kv1.5 (40.1 ± 1.2, *n* = 6). The Kv1.2–6 subunits appeared not different in KA vs. naïve samples (CT values naïve: see above; KA: Kv1.2, 34.8 ± 0.9, *n* = 18, *p* = 0.72; Kv1.3, 39.4 ± 0.6, *n* = 14, *p* = 0.1; Kv1.4, 39.8 ± 0.8, *n* = 15, *p* = 0.67; Kv1.5, 41.3 ± 0.7, *n* = 12, *p* = 0.57; Kv1.6, 36.2 ± 0.7, *n* = 17, *p* = 0.94). However, Kv1.1 mRNA was present at 6.1-fold higher levels in KA vs. naïve samples (CT KA: Kv1.1, 33.3 ± 0.7, *n* = 22; *p* < 0.05; Figure 4B). These results are consistent with the tissue level analysis; the Kv1.1 upregulation was even more pronounced.

The above described qPCR methods do not allow correlation of mRNA levels with cell physiology. Furthermore, harvesting of components from other cell types including synaptic terminals could distort the analysis. Therefore, we additionally probed all Kv1 channels using the true SC RT-qPCR method. Since the pipette solution required for RT-qPCR is of unphysiological contents, we developed a method combining gramicidin-perforated recordings with subsequent membrane rupture and cytosol harvesting for SC RT-qPCR (Figures 4C,D). Using this technique, Kv1.1–6 subunits were present in the following relative abundance order in naïve DG cells (CT values): Kv1.2 (39.2 ± 1.7, *n* = 14) > Kv1.3 (39.6 ± 0.9, *n* = 11) > Kv1.4 (40.6 ± 0.7, *n* = 12) > Kv1.6 (41.1 ± 1.1, *n* = 11) > Kv1.5 (43.2 ± 1.0, *n* = 9) > Kv1.1 (43.6 ± 1.0, *n* = 19). Consistent with our results from the pearl method, levels of Kv1.2–Kv1.6 mRNA were similar in naïve and KA cells (CT KA: Kv1.2, 39.0 ± 0.9, *n* = 22, *p* = 0.83; Kv1.3, 40.6 ± 0.8, *n* = 20, *p* = 0.66; Kv1.4, 42.6 ± 0.9, *n* = 21, *p* = 0.1; Kv1.5, 43.3 ± 1.0, *n* = 16, *p* = 0.97; Kv1.6, 39.5 ± 1.2, *n* = 16, *p* = 0.36). However, with the SC RT-qPCR technique, the Kv1.1 difference was even further pronounced (10.4-fold in KA cells; CT KA: Kv1.1, 40.2 ± 0.8, *n* = 22; KA vs. naïve, *p* < 0.05; Figure 4C). Thus, despite the lower yield of cDNA from SC (and corresponding higher variability), compared to pearl and tissue methods, the Kv1.1 upregulation in KA cells was robust. The combination of perforated and RT-PCR techniques allowed us to test whether Kv1.1 subunit quantity is directly related to response delays (pooling KA and naïve cells). Indeed, response delays of DG cells correlated with the amount of Kv1.1 mRNA (*r* = 0.56, *n* = 32, *p* < 0.01; Figure 4D). We did not detect such a relation with the other Kv1 subunits (CT vs. delay: Kv1.2, *p* = 0.82, *n* = 18; Kv1.3, *p* = 0.95, *n* = 17; Kv1.4, *p* = 0.67, *n* = 16; Kv1.5, *p* = 0.69, *n* = 14; Kv1.6, *p* = 0.45, *n* = 17).

From our data it is not possible to judge the exact stoichiometry of delay channel subunits; DTX-insensitive subunits could be part of a DTX-sensitive channel (Ruppersberg et al., 1990). A hint for potential co-regulation of genes can be obtained via the z-score on (co-) variances (see Methods). While Kv1.2, 1.3- and 1.5 subunits showed no correlation in z-scores with Kv1.1 (Kv1.2, *p* = 0.13, *n* = 21; Kv1.3 *p* = 0.92, *n* = 16, Kv1.5 *p* = 0.44, *n* = 13), the z-scores of Kv1.6 and Kv1.1 were strongly correlated (*p* < 0.0001). However, also the z-score of Kv1.4 correlated with Kv1.1 (*p* < 0.01). In combination with the above detected elevation of Kv1.6 subunits in KA cells (3.2-fold but not significant), the z-scores could nevertheless indicate that Kv1.6 is co-upregulated with Kv1.1 (see Discussion). In summary, the combined results of our different RT-qPCR analyses provide strong evidence that the increase in AP delay of DG cells is mediated via an upregulation of Kv1.1 subunit transcription.

### Expression of Kv1.1 proteins is more abundant in the KA-injected hippocampus

**Figure 5:**
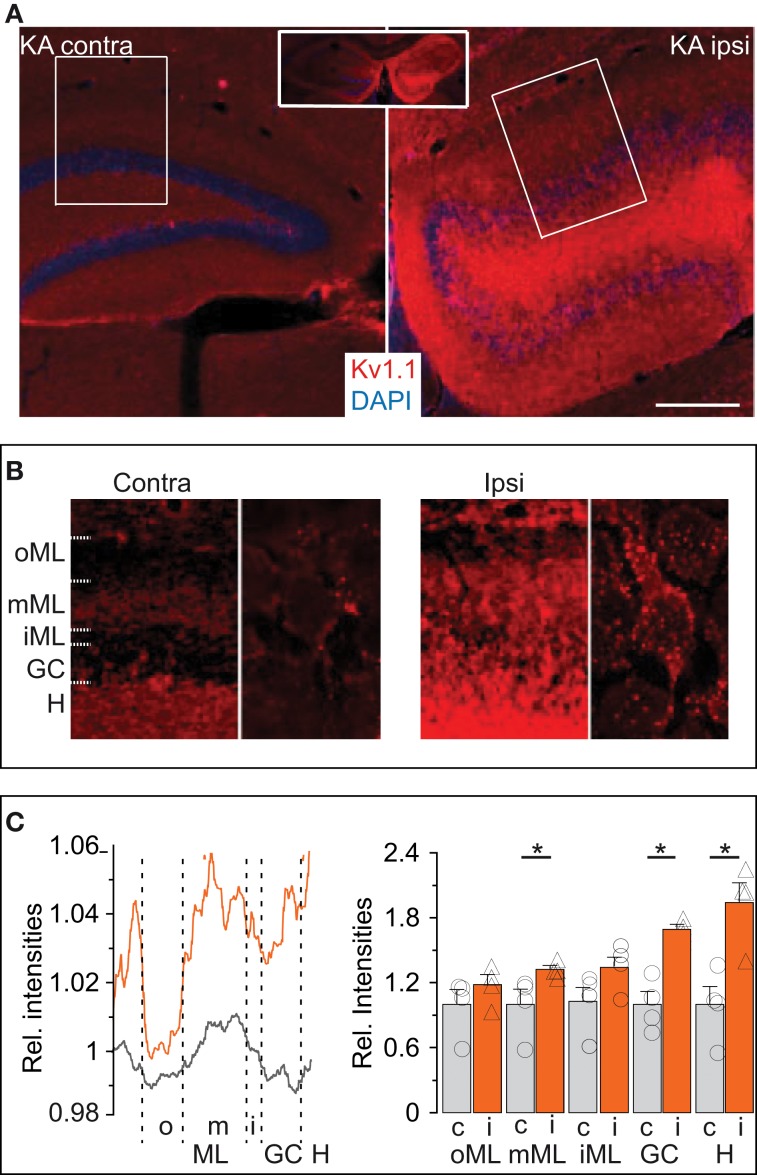
**Expression of Kv1.1 proteins is more abundant in the KA-injected hippocampi. (A)** Overview (*inset*, both hemispheres) of immunofluorescence labeling of Kv1.1 channel proteins (*red*) strongly enhanced in the (*ipsilateral, Ipsi*) KA-injected hippocampus compared to the non-injected (*contralateral, Contra*) side. Sections were co-labelled with DAPI (*blue*). *Scale bar*, 500 μm. **(B)** Magnified regions of interest acquired with identical microscope settings (*left panels* of *Contra and Ipsi*, ROIs boxed in **(A)** respectively; *right panels*, further magnified single cells, respectively). Contralateral Kv1.1 signal intensities were larger in the hilus (*H*) and middle molecular layer (*mML*, see Results) compared to the DG cell soma layer (*GC*) as well as outer and inner ML (*oML, iML,* respectively). Ipsilateral Kv1.1 signals also formed a band in the mML, but in addition showed strong Kv1.1 labeling in the GC, iML and H. At higher magnification, contralateral DG cells show little Kv1.1 in the GC layer (*right panels in Contra and Ipsi*). However, the KA side shows abundant Kv1.1 punctae decorating DG somata which may belong to somatodendritic membranes of DG cells or backsprouted mossy fibers (*Ipsi*, *right panel*). **(C)** Relative quantification of Kv1.1 fluorescence intensities by line profiles (*left panel*) or surface areas (*right panel*) from ROIs as in **(B)**. The line profile (*left panel*) shows a distinctive band in the mML contra- and ipsilateral. The relative amount of Kv1.1 signal is enhanced ipsilateral in almost all dentate regions.

We performed immunofluorescence labeling to obtain additional information on the abundance and distribution of Kv1.1 proteins in hippocampi of KA mice, starting with the molecular layer (ML). In the contralateral hippocampus not affected by sclerosis, a band of higher Kv1.1 labeling was observed in the middle ML (mML) of the dentate gyrus (Figures 5A,B,D). This band corresponds well to the previously demonstrated Kv1.1-containing presynaptic terminals of the medial perforant path (Wang et al., 1994; Monaghan et al., 2001). The hilus was also intensively stained with Kv1.1 antibodies (Figures 5A,B), again indicating the accumulation of Kv1.1 in axonal compartments, this time of the DG cells themselves. The somata of contralateral DG cells displayed very little Kv1.1 immunostaining (Figure 5B). In the dentate gyrii of the KA-injected (ipsilateral) side, Kv1.1 protein expression was overall enhanced (Figure 5A). Higher levels of Kv1.1 protein were not only apparent in the hilus and mML (relative signal intensity KA: mML, 1.32 ± 0.04; *n* = 4; *p* < 0.05; hilus, 1.94 ± 0.18; *n* = 4; *p* < 0.05) but also in the dispersed DG soma layer (relative signal intensity GC: KA-injected, 1.69 ± 0.5; *n* = 4; *p* < 0.05; Figures 5A–C). At higher magnification, somatodendritic membranes of DG cells in KA-injected hippocampi appeared more intensively decorated with Kv1.1 protein (Figure 5B). However, this labeling could correspond to sprouted mossy fibers (Suzuki et al., 1997). Irrespectively of the subcellular location, these results are consistent with our electrophysiological and RT qPCR analyses and demonstrate that epilepsy-challenged DG cells confirm a marked increase in Kv1.1 protein.

### Response delay regulation of DG cells *in vitro* is reversible

**Figure 6:**
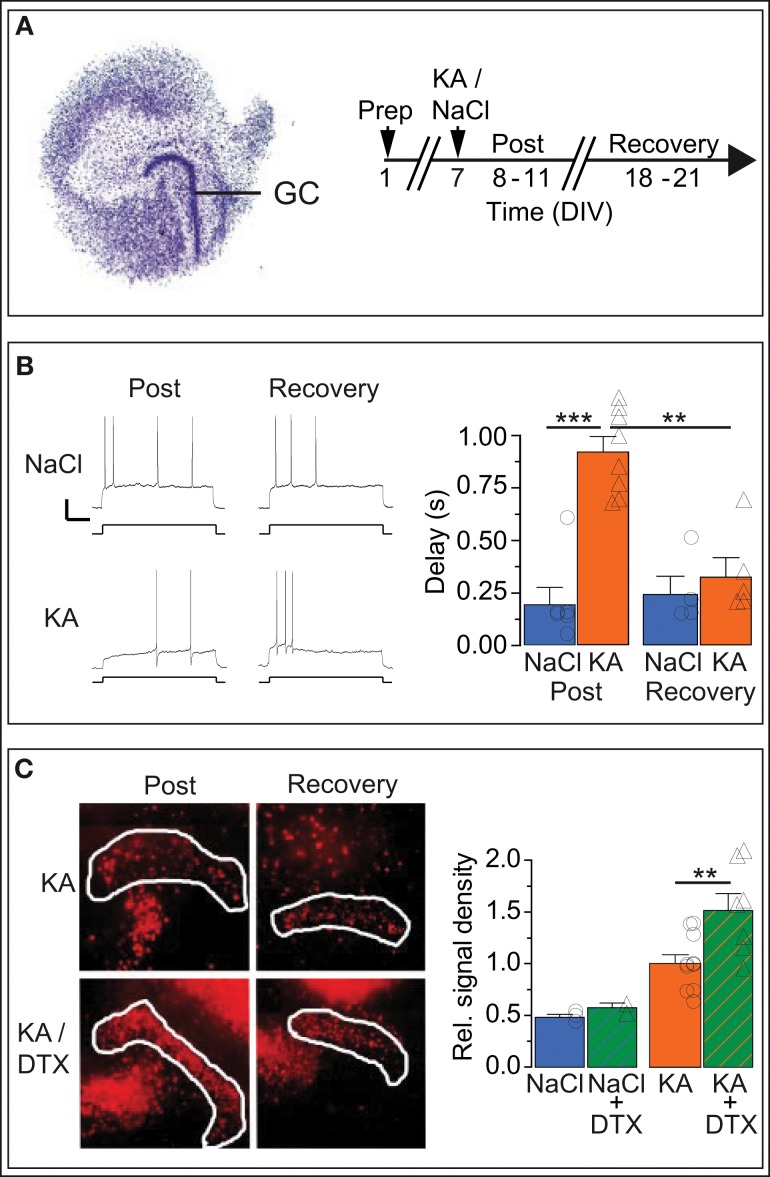
***In vitro* KA model: Kv1.1 channel upregulation in DG cells is reversible and neuroprotective. (A)** Organotypic hippocampal slice cultures (OHCs) prepared from P2 mouse pups were treated after 7 days *in vitro* (DIV) either with normal medium (*NaCl*) or with 15 μM kainate (*KA*). Time-matched results were pooled into two periods: the acute *post-KA* (or *post-NaCl)* period (8–11 DIV) and the *recovery* period (18–21 DIV). **(B)** The AP response delays of cultured DG cells were strongly enhanced in KA vs. control slices during the acute period (compare *upper* and *lower Post-*traces, and *blue Post-NaCl* and *orange Post-KA* bars, respectively). Importantly, during the recovery period, the response delays of KA-incubated slices returned to small NaCl-like values (compare *upper* and *lower Recovery* traces and *orange Post-KA* and *orange Recovery-KA* bars, respectively). *Scale bars*, 20 mV, 0.5 s. Current steps: NaCl, 70 pA; KA, 40 pA. **(C)** Staining with propidium iodide (PI), a cell death marker, showed that KA induced widespread cell death of hippocampal cells (*left, Post-KA subpanel*), which was quantified in the DG cell layer (*white border*, see Methods). The addition of Kv1 blocker DTX in the acute phase only mildly affected cell death under control conditions (*NaCl, NaCl/DTX*) but led to a massive increase in cell death in the DG cell layer (*left panel, Post-KA/DTX subpanel, and right panel*). These results indicate that the presence of functional Kv1 channels has a neuroprotective effect for DG cells.

Ion channel plasticity of DG cells could represent an intrinsic, homeostatic (i.e., reversible) output adaptation to protect DG cells from excitotoxic cell death. As the epilepsy of KA-injected mice is not reversible, we turned to organotypic hippocampal slice cultures (OHCs) to test whether hyperexcitability-induced upregulation of response delays reverses when the excitotoxic stimulus is discontinued. We used a transient KA incubation as a hyperexcitability stimulus because this protocol constitutes an established *in vitro* seizure model associated also with cellular symptoms of TLE (Routbort et al., 1999; Bausch and McNamara, 2004; Tinnes et al., 2011; Chai et al., 2013). We incubated the OHCs on DIV 8 with medium containing either no drug (i.e., NaCl) or 10 μM KA for 1 day (Figure 6A). Then we performed perforated recordings of OHC DG cells first in the acute phase after KA or NaCl incubation (“post-KA” or “post-NaCl,” respectively) or in the recovery period (Figures 6A,B).

Although the *in vitro* KA model clearly constitutes a different condition compared to the *in vivo* KA model, the *R*_in_ of OHC DG cells decreased similarly in the days after KA treatment (*R*_in_: post-NaCl, 765.3 ± 94.1 MΩ, *n* = 6; post-KA, 318.3 ± 48.0 MΩ, *n* = 6; post-KA vs. post-NaCl *p* < 0.01). As expected for DG cells in culture which have less dendritic arborization (Lossi et al., 2009), *R*_in_ values of OHC DG cells were higher compared to acute brain slices (see Methods). With *R*_in_ values of immature DG cells being above 1 GΩ in acute slices, one could argue DG cells of OHCs may represent a different maturation state compared to acute slices (Schmidt-Hieber et al., 2004). However, the neurogenic potential is low in OHCs (Namba et al., 2007) and further reduced after KA application (Sadgrove et al., 2005; Gerlach, pers. commun.), making it very unlikely that any of these recordings were from newly generated DG cells. Consistently, the AP response patterns of OHC DG cells (Figure 6B) were very different from those of immature DG cells (Schmidt-Hieber et al., 2004; Häussler et al., 2012). Importantly, the AP response delays of DG cells increased as *in vivo* in the days following KA treatment *in vitro* (delays: post-NaCl, 191 ± 84 ms, *n* = 6; post-KA, 924 ± 74 ms, *n* = 8; *p* < 0.001; Figure 6B). As hypothesized for a homeostatic process, the increased delays of OHC post-KA cells returned to control-like, low values when the hyperexcitation was discontinued (delays: KA recovery, 323 ± 94 ms, *n* = 5; post-KA vs. KA recovery, *p* < 0.001; Figure 6B). Similarly, *R*_in_ values also recovered (*R*_in_: NaCl recovery, 597.3 ± 153.0 MΩ, *n* = 4; KA recovery, 516.6 ± 33.8 MΩ, *n* = 5). To detect potentially conflicting time-dependent effects in the OHCs, we also examined time-matched slices incubated with NaCl, but these did not show such changes in the same period (delays: post-NaCl see above; NaCl recovery, 240 ± 89 ms, *n* = 4; Figure 6B). In some examples we also probed the Kv1.1 mRNA abundance of OHC DG cells via SC RT-qPCR (not shown). Consistent with our data from the *in vivo* model, Kv1.1 mRNA was increased in post-KA vs. post NaCl (CT: post-NaCl, 42.4 ± 0.5, *n* = 5; post-KA, 37.4 ± 0.7, *n* = 5; *p* < 0.01) and returned to lower values in the recovery period (CT: KA recovery, 41.6 ± 2.2, *n* = 4). Also *in vitro* the response delay and Kv1.1 mRNA abundance correlated on the single cell level (*p* < 0.05). Thus, these results demonstrate the capacity of DG cells to reversibly regulate their response speed in reaction to conditions of hyperexcitability.

We additionally used the OHC model to test the neuroprotective potential of Kv1 channels for DG cells. To this end, cell death was evaluated via PI signal intensity with and without application of DTX during and after KA incubation. As expected, KA incubation *in vitro*, led to massive cell death in OHCs. Specifically, the PI labeling was enhanced in the DG cell layer after KA vs. control OHCs (PI relative to post-KA: post-NaCl, 0.48 ± 0.03, *n* = 3; post-KA, 1.00 ± 0.09, *n* = 10; *p* < 0.01; Figure 6C). However, when blocking Kv1 channels with DTX, cell death was much stronger in the DG cell layer (PI relative to post-KA, 1.00 ± 0.85, *n* = 10; post-KA/DTX, 1.51 ± 0.17, *n* = 7; *p* < 0.01; Figure 6C). In contrast, in control OHCs, DTX had only minor effects (PI relative to post-KA: post-NaCl, 0.48 ± 0.03, *n* = 3; post-NaCl/DTX, 0.58 ± 0.05, *n* = 2; Figure 6C). The results of this section are consistent with the hypothesis that the upregulation of response delays constitutes a homeostatic mechanism able to protect the DG cells from toxic hyperexcitation under epileptic conditions.

## Discussion

The main result of the present study is that a DTX-sensitive delay current (I_D_) mediated by Kv1 channels controls the AP response delay of DG cells. Under conditions of epileptic hyperexcitability the delay is scaled up considerably via transcriptional upregulation of Kv1.1 subunits. In addition to local signal processing in subcellular compartments such as dendrites and spines (Yuste et al., 1994; Krueppel et al., 2011), neurons need cell-wide scaling mechanisms to adjust their input/output function in a homeostatic manner in particular during conditions of chronic hyperexcitation (Turrigiano and Nelson, 2000; Seeburg and Sheng, 2008). Our results suggest that the I_D_ regulation of DG cells is such a homeostatic mechanism of intrinsic plasticity and that it occurs via regulation of Kv1.1 expression which constitutes an anti-epileptic mechanism of DG cells. Indeed, mice lacking Kv1.1 channels develop epilepsy (Smart et al., 1998; Wenzel et al., 2007).

### The function of Kv1 channels in DG cells

Previous studies on DG cells and other cell types have shown that DTX-sensitive Kv1 channels have a defined task in shaping the AP of presynaptic compartments in the submillisecond range (Geiger and Jonas, 2000; Kole et al., 2007; Foust et al., 2011). Consistent with this task, respective Kv1 channels are mainly targeted toward axonal membranes (Sheng et al., 1994; Wang et al., 1994; Rhodes et al., 1997; Monaghan et al., 2001; Wenzel et al., 2007). While some Kv1 channels may also be expressed in dendrites (Sheng et al., 1994; Wang et al., 1994; Veh et al., 1995), somatic membranes of DG cells were reported devoid of DTX-sensitive currents (Riazanski et al., 2001). Consistently, Kv1.1 labeling in somata of contralateral DG cells is very weak. However, the effects of DTX in our somatic recordings suggest that either the Kv1 channels which are expressed in axonal compartments can efficiently interfere with somatodendritic subthreshold signal integration, or that functional Kv1 channels are expressed in DG cells dendrites (Raab-Graham et al., 2006; Metz et al., 2007). Considering the Kv1 channel distribution (see above), a dendritic role for Kv1 channels appears less likely; the subthreshold Kv channels which rule this domain are mostly of the Kv4 family (Schoppa and Westbrook, 1999; Shibata et al., 2000; Bekkers and Delaney, 2001; Rhodes et al., 2004). However, DG cells are electrotonically compact and with respect to somatodendritic signal integration and AP initiation in the proximal axon, DG cells may act as single computational unit (Schmidt-Hieber et al., 2007; Schmidt-Hieber and Bischofberger, 2010). In other cell types, the I_D_ can both improve and weaken precision of AP timing (Cudmore et al., 2010; Higgs and Spain, 2011). Our data show that the I_D_ of DG cells weakens absolute AP precision but relative to AP delay, precision (CV) remains relatively constant with I_D_ scaling.

### The Kv1 channels underlying I_D_ and Kv1.1 plasticity during hippocampal epilepsy

The Kv subunits underlying the I_D_ have not been unequivocally identified. Since homomeric channels of Kv1.1, Kv1.2, and Kv1.6 subunits are DTX-sensitive (Stuhmer et al., 1989; Grupe et al., 1990; Grissmer et al., 1994), various combinations of (only) these subunits have been considered to mediate I_D_ (Golding et al., 1999; Bekkers and Delaney, 2001; Dodson et al., 2002; Glazebrook et al., 2002; Guan et al., 2006; Miller et al., 2008). However, in principle, any combination with a single DTX-sensitive subunit in a heterotetramer could mediate the I_D_ (Stuhmer et al., 1989; Ruppersberg et al., 1990; Hatton et al., 2001). Our data demonstrate that the increase in I_D_ of DG cells is mediated by an upregulation of Kv1.1 subunit expression. Not only did we observe Kv1.1 mRNA upregulation on different PCR analysis levels, but we recorded a direct correlation between Kv1.1 mRNA amount and response delays of DG cells on the single cell level. In addition, we detected an increase in Kv1.1 protein expression. Yet, it is still possible that only the relative amount of Kv1.1 subunits increased in assembled DTX-sensitive channels. Generally, subunit composition could affect the membrane targeting (Manganas and Trimmer, 2000). Specifically, a higher Kv1.1 content in Kv1 channels is known to shift the voltage-dependence to more hyperpolarized potentials (Stuhmer et al., 1989; Grupe et al., 1990; Grissmer et al., 1994), which we indeed observed. In OHCs, we detected increased cell death with Kv1 channel inhibition. Although we cannot exclude that DTX itself triggered a homeostatic Kv1 channel expression, it appears more likely that the KA-induced hyperexcitation (Routbort et al., 1999; Bausch and McNamara, 2004) was the main factor increasing I_D_ and Kv1.1. Without KA, DTX did not increase I_D_ in the same time period, while without DTX; KA did; these data suggest that the destructive effect of DTX was mainly via inhibition of neuroprotective Kv1 channels. As for all *in vitro* experiments, OHCs cannot be directly compared to the *in vivo* situation (despite the similarity of I_D_ and Kv1.1 upregulation). However, the OHC experiments display the potential reversibility of I_D_ scaling in DG cells. Concerning the channel stoichiometry, we do not know the exact subunit composition of native complexes. The abundance of mRNA in our qPCR data would favor Kv1.2 and possibly Kv1.6. Consistent with this hypothesis, except for a small population of Kv1.4-comprising channels, previously discovered native Kv1.1-containnig complexes always contained Kv1.2 and probably contained Kv1.6 and auxiliary subunits (Koch et al., 1997; Shamotienko et al., 1997; Coleman et al., 1999; Wang et al., 1999). Other Kv channels such as A-type channels also exist in DG cells (Sheng et al., 1992; Rhodes et al., 1997, 2004; Riazanski et al., 2001; Ruschenschmidt et al., 2006) but in view of the short AP delay remaining in DTX, their relative influence on the AP delay of DG cells appears small. We have not investigated the upstream molecular events of transcriptional Kv1.1 regulation; these processes could be similar to other activity-dependent plasticity mechanisms (Fan et al., 2005; Misonou et al., 2006), perhaps including relocation of axonal channels (Grubb and Burrone, 2010) and a replay of developmental programs (Riazanski et al., 2001; Brewster et al., 2002; Mongiat et al., 2009).

### Heterogeneous functional phenotypes of mature DG cells

On average, outer DG cells closer to the ML possessed larger response delays. One reason for such a functional gradient in the DG cell population could be adult neurogenesis which continuously adds newborn cells from the subgranular zone (Overstreet-Wadiche and Westbrook, 2006). As immature DG cells possess smaller dendritic trees and high *R_in_* values (Schmidt-Hieber et al., 2004; Häussler et al., 2012), a gradient in these parameters within the DG cell population is expected (Liu et al., 2000; Wang et al., 2000). However, after ~4 weeks *R*_in_ values have been reported to remain stable indicating that mature DG cells are functionally relatively homogenous (Van Praag et al., 2002). We did not record from immature neurons (see Results) but nevertheless detected the described heterogeneity in response delays. Thus, in addition to the early maturation of morphology and passive properties (Wang et al., 2000; Overstreet-Wadiche and Westbrook, 2006; Mongiat et al., 2009), changes in response delays contribute to a functional heterogeneity among mature DG cells, which may improve the dentate encoding capabilities (Padmanabhan and Urban, 2010).

### The relevance of I_D_ regulation for hippocampal function

With respect to the functioning of the hippocampal network, our results are relevant in several ways. First, the low firing rates of DG cells are thought to be crucial to obtain a sparse representation during hippocampal processing (Treves and Rolls, 1992; Jung and McNaughton, 1993). We revealed I_D_ regulation as a powerful intrinsic tool of DG cells to homeostatically achieve the sparse firing. Finally, the dentate network is thought to implement a temporal winner-take-all mechanism where fast responders silence their slower neighbor DG cells (De Almeida et al., 2009). The slow responders we observed in the outer DG cell layer and particularly in epileptic animals are likely to be the “losers” in this race to threshold and also miss the timing relative to hippocampal oscillations (Buzsaki, 2002; Lin et al., 2012). However, since the response speed ratio of inner to outer DG cells is roughly maintained in epileptic animals, it is possible that dentate function is maintained during I_D_ scaling. Alternatively, or in addition, the I_D_ regulation may be a molecular mechanism for the “early retirement” of DG cells (Alme et al., 2010). Accordingly one may formulate: faced with epileptic excitotoxicity, DG cells opt to retire, lose and survive, rather than to win and die.

## Author contributions

Florian Kirchheim performed all presented electrophysiological, pharmacological, molecular biology (SC RT-qPCR), and immunocytochemistry experiments, as well as KA injections and analyzed the respective results; Stefanie Tinnes prepared the OHCs, applied their treatments and analyzed the PI data with Florian Kirchheim; Carola A. Haas supervised Stefanie Tinnes; Michael Stegen performed initial electrophysiological and pharmacological experiments and assisted supervising Florian Kirchheim; Jakob Wolfart invented and led the study, supervised all experiments and analyses, and wrote the article.

### Conflict of interest statement

The authors declare that the research was conducted in the absence of any commercial or financial relationships that could be construed as a potential conflict of interest.
