# Supplementary material for: Regulation of action potential delays via voltage-gated potassium Kv1.1 channels in dentate granule cells during hippocampal epilepsy
Source: Front Cell Neurosci. 2013 Dec 5;7:248. doi: 10.3389/fncel.2013.00248 (PMC3852106; doi:10.3389/fncel.2013.00248)
Supplement: Supplementary file 1 [file DataSheet1.PDF]

SUPPLEMENTARY MATERIAL

Supplementary Methods: Electrophysiology

Before we used potassium methylsulfonate (KMe)-based pipette solution for gramicidin-perforated patch-clamp recordings as described in the main text, we employed a more common potassium gluconate (KGlu)-based pipette solution. This KGlu solution contained (in mM): 135 KGlu, 20 KCl, 10 HEPES, 0.1 EGTA, 2$MgCl_2$, 2 Na_2ATP, and 0.2% biocytin (pH 7.35). Although KGlu solutions is widely used for gramicidin perforation, we found high rates of spontaneous membrane ruptures distorted pharmacological effects in initial experiments. To ameliorate the gramicidin-perforated technique and obtain more stable recordings of the currents of interest we systematically compared perforation stability with KGlu- and KMe-based pipette solutions (**Figures S1A-C**). Pipette tip resistance (R_{pip}), seal resistances (R_{seal}) and series resistance (R_{ser}) were not different in these recordings (R_{pip}: KMe, 5.1 ± 0.2 MΩ, n = 13; KGlu, 5.2 ± 0.1 MΩ, n = 14; p = 0.19; R_{seal}: KMe, 4.4 ± 0.4 GΩ, n = 95; KGlu, 8.2 ± 2.9 GΩ, n = 12; R_{ser}: KMe, 32.8 ± 0.6 MΩ, n = 91; KGlu, 33.7 ± 1.0 MΩ, n = 12). Following seal formation, perforation usually occurred within 5-30 min and was accompanied by a R_{in} drop towards values comparable to whole-cell conditions (see Young et al., 2009 in main text) (**Figure S1A**). After variable time periods, spontaneous membrane rupture and conversion to standard whole-cell configuration occurred and were accompanied by a change in kinetics and amplitude of capacitive currents (**Figure S1A**). To verify that membrane rupture coincided with described electrophysiological symptoms, Alexa488 hydrazide (5 µM, n = 6) was added to the pipette solution and fluorescence was monitored during recordings (**Figure S1A**). In summary, KMe-based recordings yielded stable perforated conditions over 23.7 ± 4.1 min (time from seal to membrane rupture; n = 13), while KGlu-based solutions often led to spontaneous membrane ruptures within a few minutes (2.7 ± 0.7 min, n = 14; **Figures S1B,C**).

Supplementary Methods: Morphology and immunocytochemistry

The location, morphology, and immunolabeling with DG cell marker Prox1 were verified for a subset of DG cells (**Figure S1D**) (24 of 95) and were similar as previously described (Young et al., 2009). Briefly, brain slices were fixed in PFA, washed and treated 30 min with blocking solution (see Methods). Thereafter slices were incubated with rabbit polyclonal anti-Prox1 antibody (1:1000, Chemicon, Temecula, CA, US; in 0.1% Triton plus 1% NGS). Following washing steps, avidin-D-coupled fluorochrome (FITC, or Alexa546, 1:500, Vector Laboratories) and a secondary anti-rabbit antibody conjugated with Alexa Fluor-488 (1:200, Invitrogen, Darmstadt, Germany) were added. After repeated washing, slices were mounted on gelatine-coated glass slides (Langenbrink) using fluorescence mounting medium (DAKO, Glastrup, Denmark) or ProLong gold antifade reagent (Invitrogen). Cell morphologies and immunosignals were visualized with a conventional epifluorescence microscope (Axioplan 2, x20/0.75 objective, Zeiss) or a Fluoview Fv10i confocal microscope with an UPSALP60xO objective and Fv10i imaging software (Olympus, Hamburg, Germany). Cell reconstructions were obtained by collecting 3-D image stacks from 0.5 to 0.9 µm thin optical slices (1024 x 1024 lines, Kalman-filtered). Filter settings were adjusted to FITC (473 nm excitation, 519 nm emission, 490 nm bandpass filter) and Cy3 (559 nm excitation, 567 nm emission, 570 nm bandpass filter).

Supplementary Methods: RT-qPCR

In order to establish the pearl RT-qPCR method using a previously determined increase of inward rectifier K^+ type 2 (Kir2) channels (Young et al., 2009), we probed the abundance of Kir2.1 mRNA in KA vs. naïve cells using the following primer (Primer-Blast; forward primer: 5´-TCTCACTTGCTTCGGCTCAT-3´, reverse primer: 5´-ACTTGTCCTGTTGCTGGTACA-3´. Consistent with our previous results (Young et al., 2009), Kir2.1 mRNA was strongly elevated in KA compared to naïve cells, i.e. detection thresholds were reduced (CT values: naïve, 41.6 ± 0.5, n = 6; KA, 38.9 ± 0.7, n = 7, p < 0.05). Due to the approximate doubling of cDNA per PCR cycle, this difference amounts to a factor of 6.6 ± 0.9. For Kv1 subunit analysis, the following primers were used in qPCRs: Kv1.1 (forward primer:

5´GAGAATGCGGACGAGGCTTC-3´, reverse primer: 5´-CCGGAGATGTTGATTACTACGC-3´), Kv1.2 (forward primer 5´-GGTTGAGGCGACCTGTGAAC-3´, reverse primer: 5´-TCTCCTAGCTCATAAAACCGGA-3´), Kv1.3 (forward primer: 5´-GGAGACCTTGTGCATCATCTG-3´, reverse primer 5´-CCCATTACCTTGTCGTTCAGC-3´), Kv1.4 (forward primer: 5´-GTAGCCGTGGAAGTAGAAGGA-3´, reverse primer: 5´-AGGTCTGTGTACGAACACCCA-3´), Kv1.5 (forward primer: 5´-CAATCAGGGGTCGCACTTCTC-3´, reverse primer: 5´-ACAGTCGTCATAGT-GACTACTGC-3´; Kv1.6 (forward primer: 5´-GAGTCCGTTTCTTTGACCCCT-3´, reverse primer: 5´-GGCGACCTCCAGATTGATAGTA-3´), GAPDH (forward primer: 5´-AGGTCGGTGTGAACGGATTTG-3´, reverse primer: 5´-GGGGTCGTTGATGGCAACA-3´; Kir2.1 (forward primer: 5´-TCTCACTTGCTTCGGCTCAT-3´, reverse primer: 5´-ACTTGTCCTGTTGCTGGTACA-3´) of which Kv1.1, Kv1.2, and Kv1.6 were obtained from Primer Bank (http://pga.mgh.harvard.edu/primerbank) and the remaining primers were designed with Primer-Blast (http://www.ncbi.nlm.nih.gov/tools/primer-blast/).

FIGURE S1 of Supplementary Material

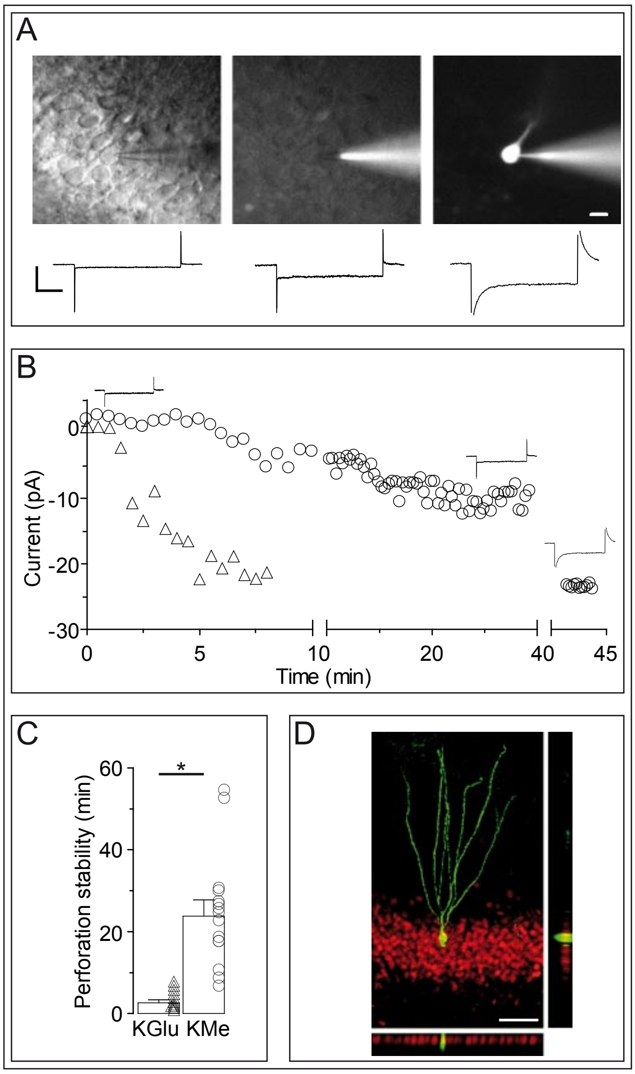

FIGURE S1 (Supplementary Material) Methylsulfonate- and gluconate-based gramicidin-perforated recordings of dentate gyrus granule (DG) cells.
(A-C) The stability of gramicidin-perforated recordings of DG cells was affected by the pipette solution. When we applied the commonly used gluconate-based pipette solution (*triangles*), the attached (*A, left panel*) and or perforated configuration (*A, middle panel*) were often subject to spontaneous membrane rupture as visualized with Alexa488 entering the cell (*A, right panel*) and measured with simultaneous capacitance increase (current traces below and in *B*, holding potential -70 mV). *Scale bars*, 10 µm, 15 pA, 20 ms. In contrast, when we used KMeSO4 (*circles*) for gramicidin perforation, the perforated configuration was on average stable for longer periods (*C*). **(D)** Immunohistochemical staining of the DG cell layer with marker Prox1 (*red*) and a recorded DG cell co-labeled with biocytin (*green*).
